# Development and validation of a nomogram for evaluating the incident risk of carotid atherosclerosis in patients with type 2 diabetes

**DOI:** 10.3389/fendo.2023.1131430

**Published:** 2023-02-16

**Authors:** Xiao Feng, Liying Ren, Yuping Xiang, Yancheng Xu

**Affiliations:** ^1^ Laboratory of Endocrine Department, Zhongnan Hospital of Wuhan University, Wuhan, China; ^2^ Key Laboratory of Hepatitis C and Immunotherapy for Liver Disease, Peking University People’s Hospital, Peking University Hepatology Institute, Beijing, Beijing, China

**Keywords:** diabetes, carotid atherosclerotic, predictive model, nomogram, diabetic complication

## Abstract

**Introduction:**

The purpose of this study was to evaluate the clinical characteristics of carotid atherosclerotic disease in patients with type 2 diabetes mellitus, investigate its risk factors, and develop and validate an easy-to-use nomogram.

**Methods:**

1049 patients diagnosed with type 2 diabetes were enrolled and randomly assigned to the training and validation cohorts. Multivariate logistic regression analysis identified independent risk factors. A method combining least absolute shrinkage and selection operator with 10-fold cross-validation was used to screen for characteristic variables associated with carotid atherosclerosis. A nomogram was used to visually display the risk prediction model. Nomogram performance was evaluated using the C index, the area under the receiver operating characteristic curve, and calibration curves. Clinical utility was assessed by decision curve analysis.

**Results:**

Age, nonalcoholic fatty liver disease, and OGTT3H were independent risk factors associated with carotid atherosclerosis in patients with diabetes. Age, nonalcoholic fatty liver disease, smoke, HDL-C, and LDL-C were characteristic variables used to develop the nomogram. The area under the curve for the discriminative power of the nomogram was 0.763 for the training cohort and 0.717 for the validation cohort. The calibration curves showed that the predicted probability matched the actual likelihood. The results of the decision curve analysis indicated that the nomograms were clinically useful.

**Discussion:**

A new nomogram was developed and validated for assessing the incident risk of carotid atherosclerotic in patients with diabetes; this nomogram may act as a clinical tool to assist clinicians in making treatment recommendations.

## Introduction

Globally, diabetes mellitus (DM), a group of diseases defined by chronic hyperglycemia, is becoming more prevalent. According to the International Diabetes Federation, by 2021, 537 million people aged 20 to 79 will have diabetes worldwide (1). Diabetes causes many problems, with microvascular and macrovascular consequences accounting for a significant portion of the cost of treatment for type 2 diabetes mellitus (T2DM) (2). Based on a follow-up of deaths in 10 study centers worldwide, cardiovascular disease is the chief cause of death in diabetic patients, explaining 44% of deaths in patients with type 1 diabetes and 52% of deaths in T2DM (3).

Atherosclerosis is the main pathological cause of macroangiopathy in diabetic patients and is an independent risk element for cardiovascular disease. On the other hand, patients with atherosclerosis secondary to DM are particularly susceptible to Cardiovascular and cerebrovascular diseases (CCVD) or other vascular diseases, which will significantly reduce their quality of life (4–6). As living standards improve, CCVD events are becoming younger, and mortality and disability are increasing (7). Therefore, to reduce cardiovascular and cerebrovascular diseases and their complications, early identification of patients with atherosclerotic cardiovascular disease and effective interventions are important (7). The early involved vessels in atherosclerotic lesions are carotid arteries (8). Carotid atherosclerosis disease (CAD) is associated with the severity of ischemic cardiovascular disease and also acts as a window into the status of other vascular atherosclerosis in the body (9, 10). Guoqing Huang et al. established CAD risk prediction models based on age, body mass index (BMI), diastolic blood pressure (DBP), DM, alanine aminotransferase (ALT), aspartate aminotransferase (AST), and gamma-glutamyl transpeptidase (GGT) for the normal population (11). Fengqi Guo et al. showed that the occurrence of CAD in the T2DM population is closely related to gender, age, hypertension, lipids, diabetic retinopathy, and other factors (12). We did not find the development of a clinical risk prediction model based on this special population by searching existing literature reports. Therefore, in this study, we aimed to create a practical nomogram for predicting the risk of CAD in adults with T2DM. The nomogram is a common visual presentation tool for disease risk prediction models that is simple and easy to use (13). In addition, a clear understanding of the important risk factors for CAD will provide a foundation for preventing and delaying serious complications.

## Materials and methods

### Study population

In this retrospective cross-sectional study, we collected 1049 patients with T2DM hospitalized at Zhongnan Hospital of Wuhan University from January 2015 to May 2021 according to inclusion and exclusion criteria. Those with severe missing information (more than 20% of the total) were excluded, and those with less missing information (less than 20% of the total) were filled by multiple interpolations. Patients were divided into a training cohort and a validation cohort by setting seeds in R. The inclusion criteria for participants were T2DM diagnosis based on the World Health Organization (WHO) guidelines (1999) (14). The main exclusion criteria were as follows: (1) Patients with severe infection, acute myocardial infarction, acute cerebral infarction, severe trauma, malignant tumor, surgery, and another stress state; (2) those taking lipid-lowering drugs; (3) those diagnosed with diabetes during pregnancy; (4) those without complete ultrasound measurements of the carotid artery.

**Figure 1:**
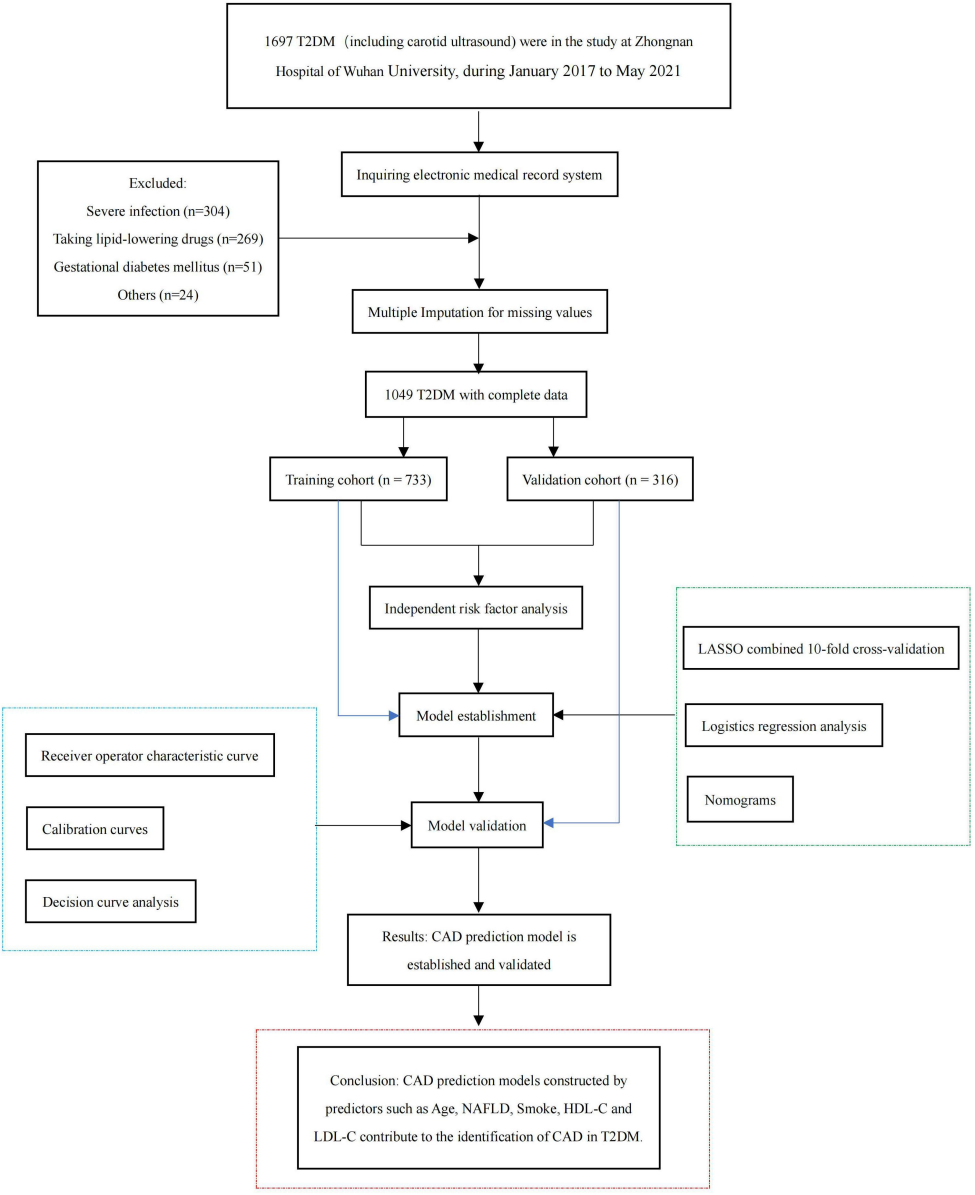
Flowchart of the participants.

The study met the ethical standards of the Declaration of Helsinki (2013) and was approved by the Ethics Committee of Zhongnan Hospital of Wuhan University (ethical approval code: 2022167K). The flow chart of the participants is shown in **Figure 1**.

### Collection of clinical data

The clinical data were obtained from medical records. Personal history (gender, age, height, body weight; smoking or drinking habits; high blood pressure, and family history of diabetes (FHD)), laboratory serological indexes (liver function, renal function, blood fat, oral glucose tolerance test (OGTT), insulin-releasing test (IRT), glycosylated hemoglobin (HbA1c), and glycosylated serum protein (GSP)), and laboratory data (carotid color ultrasonography, and abdominal ultrasonography) were obtained.

Body mass index (BMI) (kg/m2) was calculated using the formula: body mass index (BMI) (kg/m2) = height (kg)/weight2 (m2).

### Evaluation of carotid atherosclerotic disease, OGTT test and IRT test

Color Doppler ultrasound (GE Healthcare, Milwaukee, Wisconsin) was used as the diagnostic method for CAD. Experienced sonographers were blinded to all clinical information performed in the evaluation. Carotid intima-media thickness (IMT) and carotid plaque were recorded during the ultrasound examination; IMT was defined as the distance between the leading edge of the first and second echo lines of the distant arterial wall; the presence of CAP was defined as a local increase in thickness of 0.5 mm or 50% of the surrounding IMT value (15).

Subjects underwent standard OGTT and IRT tests the following morning after fasting (8-10 hours). After fasting venous blood collection for determination of fasting glucose (OGTT0H) and fasting insulin (IRT0H), patients were instructed to take 75 g of anhydrous glucose within 5 minutes, and blood samples were collected from the anterior elbow vein at intervals of 30, 60, 120 and 180 minutes for determination of plasma glucose concentrations (OGTT0.5H, OGTT1H, OGTT2H, OGTT3H) and serum insulin concentrations (IRT0.5H, IRT1H, IRT2H, IRT3H).

### Statistical analysis

The Kolmogorov-Smirnov test was used for testing the normality distribution of continuous data. Continuous data that conformed to normal distribution were expressed as “mean ± standard deviation (x ± s) “, and t-test was used for comparison between two groups; continuous data that did not conform to normal distribution were expressed as “median (lower quartile, upper quartile) “ and Mann-Whitney U test was used for comparison between groups. The frequency of dichotomous variables was performed by χ2 analysis and was expressed as “frequency (proportion) “. The independent risk factors were identified using multivariate logistic regression analyses. First, Least Absolute Shrinkage and Selection Operator (LASSO) regression was performed using the glmnet package to screen out relevant variables. Then, a multivariate logistic regression analysis was performed on the selected variables. Nomogram was built *via* the rms and regplot packages. Sensitivity and specificity were defined by receiver operating characteristic (ROC) curves drawn by the pROC software package. The calibration curves were drawn using the rms package. The rmda package was used for decision curve analysis (DCA). SPSS 18.0 (SPSS Inc, Chicago, IL, USA) and R version 4.0.3 (https://www.rproject.org/) were used for statistical analysis, and P < 0.05 or P < 0.1 was considered statistically significant.

## Results

### Clinical characteristics of the patients with T2DM

Patients were randomly divided into a training cohort (n = 733) and a validation cohort (n = 316) according to the 7:3 ratio by setting seeds in R (16). The patients’ laboratory examination findings and clinical characteristics are shown in **Table 1**. Most patients with T2DM were male (63% and 67%, respectively). The mean age of the training cohort was 56 (48, 65) years, and that of the validation cohort was 56.5 (50, 65) years. Except for the aspartate aminotransferase (AST), there were no significant differences between the other characteristics, indicating that random grouping does not produce bias. The results of the Univariate analysis are shown in **Table 2**. The mean ages of the no-CAD group (HC) and the CAD group were 53 (43,60) years and 61 (54,68) years, respectively. The two groups differed in age, NAFLD, BMI, liver function, renal function, lipids, and blood pressure.

**Table 1:** The Clinical characteristics of patients in the training and validation cohorts.

Parameter	Training cohort	Validation cohort	*p* value
	(n = 733)	(n = 316)	
Gender (%)			0.189
male	459 (63)	212 (67)	
female	274 (37)	104 (33)	
Age (years)	56 (48, 65)	56.5 (50, 65)	0.338
NAFLD (%)			0.803
No	440 (60)	193 (61)	
Yes	293 (40)	123 (39)	
Smoke (%)			0.759
No	512 (70)	217 (69)	
Yes	221 (30)	99 (31)	
Drink (%)			0.324
No	500 (68)	205 (65)	
Yes	233 (32)	111 (35)	
FHD (%)			0.313
No	712 (97)	311 (98)	
Yes	21 (3)	5 (2)	
CAD (%)			0.913
No	390 (53)	170 (54)	
Yes	343 (47)	146 (46)	
SBP(mmHg)	128 (114, 139)	126 (113, 138)	0.179
DBP(mmHg)	75 (70, 84)	75.5 (70, 83)	0.679
BMI(kg/m^2^)	24.98 (22.84, 27.1)	24.77 (22.58, 27.04)	0.352
ALT(U/L)	22 (15, 33)	21 (15, 30)	0.125
AST(U/L)	20 (16, 26)	19 (15, 24)	**0.033**
ALP(U/L)	83 (69, 100)	83 (68, 104)	0.577
BUN (mmol/L)	5.3 (4.5, 6.3)	5.4 (4.4, 6.3)	0.913
CREA(μmol/L)	64 (53.8, 75.4)	63.85 (54, 74)	0.943
UA(μmol/L)	332.6 (269.4, 405.1)	332.8 (269.72, 399.87)	0.813
Cys-c (mg/L)	0.86 (0.73, 1)	0.85 (0.71, 1.01)	0.662
CHOL (mmol/L)	4.68 (3.89, 5.42)	4.61 (3.93, 5.62)	0.656
TG (mmol/L)	1.64 (1.14, 2.58)	1.73 (1.22, 2.73)	0.228
HDL-C(mmol/L)	0.99 (0.82, 1.19)	0.98 (0.84, 1.15)	0.543
LDL-C(mmol/L)	2.69 (2.03, 3.27)	2.66 (2.13, 3.29)	0.506
GSP(μmol/L)	409.9 (323.2, 512.4)	402.85 (325.48, 491.57)	0.427
HbA1c(%)	8.85 (7.4, 10.57)	8.55 (7.1, 10.43)	0.233
OGTT0H(mmol/L)	9.46 (7.39, 12.48)	9.14 (7.31, 12.03)	0.543
OGTT0.5H(mmol/L)	13.14 (10.62, 16.03)	13.06 (10.77, 15.96)	0.634
OGTT1H(mmol/L)	16.9 (13.89, 20.17)	16.73 (13.72, 20.29)	0.695
OGTT2H(mmol/L)	18.86 ± 5.42	18.99 ± 5.62	0.721
OGTT3H(mmol/L)	16.55 ± 5.90	16.76 ± 6.35	0.626
IRT0H(uIU/ml)	11 (6.32, 16.4)	9.95 (5.94, 15.22)	0.061
IRT0.5H(uIU/ml)	17.5 (10.7, 28.9)	16.6 (10.5, 26.52)	0.369
IRT1H(uIU/ml)	26.1 (15.1, 43.2)	25.65 (14.2, 39.51)	0.284
IRT2H(uIU/ml)	33.3 (18.7, 55)	32.05 (18.78, 50.31)	0.360
IRT3H(uIU/ml)	28.6 (15.7, 48.4)	26.3 (15.7, 45.37)	0.256

*p-value indicates statistically significant.

n, number of patients; NAFLD, nonalcoholic fatty liver; FHD, family history of diabetes; CAD, carotid atherosclerotic disease; SBP, Systolic blood pressure; DBP, Diastolic blood pressure; BMI, body mass index; ALT, alanine aminotransferase; AST, aspartate aminotransferase; ALP, alkaline phosphatase; BUN, urea nitrogen; CREA, creatinine; UA, blood uric acid; Cys-c, cystatin C; CHOL, total cholesterol; TG, triglyceride; HDL-C, high-density lipoprotein; LDL-C, low-density lipoprotein; GSP, glycated serum protein; OGTT, oral glucose tolerance test; HbA1c, glycated hemoglobin; IRT, insulin-releasing test.

**Table 2:** Univariate analysis of carotid atherosclerosis.

Parameter	Overall	HC	CAD	P value
N	1049	560	489	
Gender (%)				0.768
Male	671 (64)	361(64)	310(63)	
Female	378 (36)	199(36)	179(37)	
Age	56(49,65)	53(43,60)	61(54,68)	**0.000**
NAFLD (%)				**0.065**
No	633(60)	353(63)	280(57)	
Yes	416(40)	207(37)	209(43)	
Smoke (%)				0.474
No	729(69)	395(71)	334(68)	
Yes	320(31)	165(29)	155(32)	
Drink (%)				0.440
No	705(67)	370(66)	335(69)	
Yes	344(33)	190(34)	154(31)	
FHD (%)				0.805
No	1023(98)	545(97)	478(98)	
Yes	26(2)	15(3)	11(2)	
SBP (mmHg)	127(114,139)	126(113,138)	130(115,141)	**0.011**
DBP (mmHg)	75(70,84)	76(70,85)	75(70,83)	**0.033**
BMI(kg/m^2^)	24.91(22.77,27.08)	25.14(22.96,27.11)	24.69(22.49,26.99)	**0.028**
ALT(U/L)	22(15,32)	22.5(16,35)	20(15,29)	**0.001**
AST(U/L)	19(16,25)	20(16,26)	19(15,25)	0.127
ALP(U/L)	83(68,101)	82(69,99)	83(68,103)	0.305
BUN (mmol/L)	5.3(4.5,6.3)	5.2(4.3,6.18)	5.5(4.5,6.5)	**0.001**
CREA(μmol/L)	64(54,75)	63.8(53,74)	64.4(54,77)	**0.091**
UA(μmol/L)	332.6(269.5,404.5)	337.55(272.58,411.55)	325.8(266.5,390.7)	0.148
Cys-c(mg/L)	0.85(0.72,1)	0.81(0.7,0.97)	0.88(0.75,1.04)	**0.000**
CHOL (mmol/L)	4.64(3.91,5.49)	4.69(3.95,5.46)	4.57(3.83,5.49)	0.473
TG (mmol/L)	1.67(1.15,2.61)	1.73(1.17,2.76)	1.61(1.13,2.38)	**0.014**
HDL-C (mmol/L)	0.99(0.83,1.18)	0.98(0.82,1.19)	1(0.84,1.16)	0.512
LDL-C (mmol/L)	2.68(2.06,3.28)	2.67(2.05,3.24)	2.69(2.07,3.32)	0.293
GSP(μmol/L)	407.3(324,506.5)	403(322.78,506.85)	410.7(325.1,505.8)	0.777
HbA1c (%)	8.8(7.32,10.5)	8.6(7.2,10.65)	8.9(7.4,10.3)	0.680
OGTT0H (mmol/L)	9.38(7.37,12.41)	9.29(7.21,12.46)	9.47(7.42,12.36)	0.743
OGTT0.5H (mmol/L)	13.13(10.64,16.03)	13.28(10.51,16.34)	12.99(10.78,15.72)	0.578
OGTT1H (mmol/L)	16.83(13.87,20.21)	16.86(13.61,20.58)	16.78(14.38,19.95)	0.898
OGTT2H (mmol/L)	18.9 ± 5.48	18.65 ± 5.58	19.18 ± 5.35	0.116
OGTT3H (mmol/L)	16.61 ± 6.04	16.19 ± 6.05	17.1 ± 5.99	**0.015**
IRT0H(uIU/ml)	10.7(6.19,16.1)	10.1(6.03,15.93)	11.43(6.58,16.2)	0.067
IRT0.5H(uIU/ml)	17.1(10.7,28)	16.6(10.2,27.8)	17.8(11.3,28.1)	0.204
IRT1H(uIU/ml)	25.8(14.8,42.2)	24.7(13.97,41.21)	27.5(15.8,44.1)	0.100
IRT2H(uIU/ml)	32.83(18.7,53.9)	32.3(17.4,50.82)	34.1(20.7,56.7)	**0.052**
IRT3H(uIU/ml)	28.1(15.7,47.51)	26.85(14.78,44.82)	29.1(17.2,50.7)	**0.050**

*p-value indicates statistically significant. The bold shows that P values are meaningful.

n, number of patients; NAFLD, nonalcoholic fatty liver; FHD, family history of diabetes; CAD, carotid atherosclerotic disease; SBP, Systolic blood pressure; DBP, Diastolic blood pressure; BMI, body mass index; ALT, alanine aminotransferase; AST, aspartate aminotransferase; ALP, alkaline phosphatase; BUN, urea nitrogen; CREA, creatinine; UA, blood uric acid; Cys-c, cystatin C; CHOL, total cholesterol; TG, triglyceride; HDL-C, high-density lipoprotein; LDL-C, low-density lipoprotein; GSP, glycated serum protein; OGTT, oral glucose tolerance test; HbA1c, glycated hemoglobin; IRT, insulin-releasing test.

### Independent risk factors

Based on univariate analysis (**Table 2**), we selected candidate variables with P<0.1 for inclusion in the multivariate logistic regression analysis. Independent risk factors associated with CAD in T2DM were finally identified, including age, NAFLD, and OGTT3H (**Table 3**).

**Table 3:** Multivariate logistic regression analysis.

Parameter	Coefficients	Odds ratio (95% CI)	P value
Age	0.073	1.076(1.061–1.092)	**0.000**
NAFLD	0.474	1.607(1.204–2.151)	**0.001**
SBP	0.005	1.005(0.995–1.016)	0.310
DBP	-0.002	0.998(0.981–1.016)	0.837
BMI	-0.037	0.964(0.923–1.006)	0.096
ALT	-0.002	0.998(0.992–1.004)	0.567
BUN	-0.039	0.962(0.876–1.056)	0.413
CREA	0.003	1.003(0.995–1.011)	0.509
Cys-c	0.116	1.133(0.662–1.871)	0.624
TG	-0.003	0.997(0.933–1.061)	0.929
OGTT3H	0.026	1.026(1.001–1.053)	**0.045**
IRT2H	0.002	1.002(0.996–1.008)	0.515
IRT3H	-0.002	0.998(0.991–1.004)	0.442

*p-value indicates statistically significant. The bold shows that P values are meaningful.

NAFLD, nonalcoholic fatty liver; SBP, Systolic blood pressure; DBP, Diastolic blood pressure; BMI, body mass index; ALT, alanine aminotransferase; BUN, urea nitrogen; CREA, creatinine; Cys-c, cystatin C; TG, triglyceride; OGTT, oral glucose tolerance test; IRT, insulin-releasing test.

### Construction of predictive models

**Figure 2:**
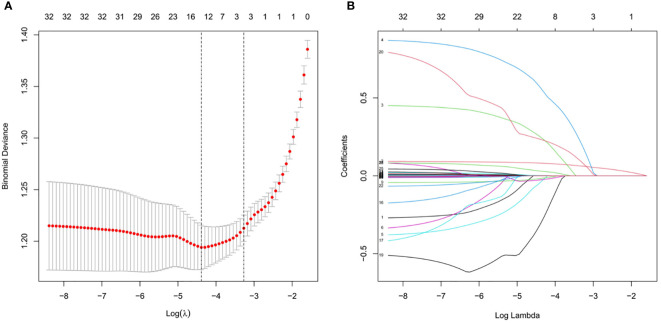
Clinical index selection using LASSO regression analysis. **(A)** Log(lambda) and partial likelihood deviations are shown, and the dotted line shown at the smallest log(lambda) represents the best predictor. **(B)** LASSO coefficients for the 32 clinical indicators. Non-zero coefficients were determined based on the best logarithm (lambda).

A total of 733 patients in the training cohort were included in the LASSO regression analysis, and 13 non-zero characteristic variables were screened (**Figure 2**). Next, to further develop predictive models for CAD, the aforementioned indicators were included in multivariate logistic regression analysis; we selected age, NAFLD, smoking, HDL-C, and LDL-C for model construction (**Table 4**).

**Table 4:** Multivariate logistic regression analysis.

Parameter	Coefficients	Odds ratio (95% CI)	P value
Age	0.093	1.097(1.078–1.117)	**0.000**
NAFLD	0.457	1.579(1.104–2.268)	**0.013**
Smoke	0.900	2.459(1.581–3.863)	**0.000**
HDL-C	-0.792	0.453(0.233–0.859)	**0.017**
LDL-C	0.316	1.371(1.128–1.673)	**0.002**

*p-value indicates statistically significant. The bold shows that P values are meaningful.

NAFLD, nonalcoholic fatty liver; HDL-C, high-density lipoprotein; LDL-C, low-density lipoprotein.

### Development of the nomogram to predict carotid atherosclerosis

**Figure 3:**
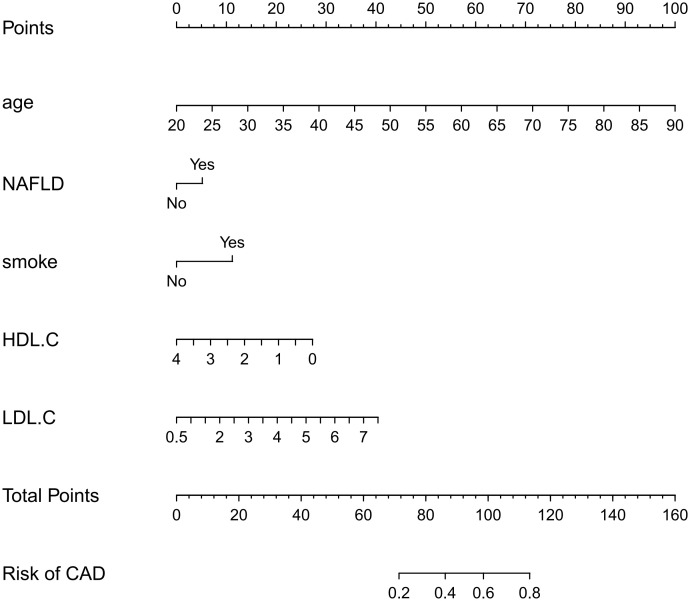
Nomogram: A Nomogram was created to determine the incidence of carotid atherosclerosis. A total score was obtained from the values of each index, and the CAD risk rate corresponding to the total score was the predicted rate of the Nomogram.

A nomogram for predicting CAD in T2DM was created based on the results of a multivariate logistic regression model (**Figure 3**). The results showed the highest risk scores (100) for age (≥90 years of age). Visualization of risk factors for CAD in T2DM can predict the risk of individual CAD. First, each unique CAD risk factor was projected upward to the first row of the scale to obtain a score for each element; then, the scores for the five risk factors were summed to get a total score. The higher the total score, the higher the CAD risk of the individual.

### Validation of the nomogram to predict carotid atherosclerosis

**Figure 4:**
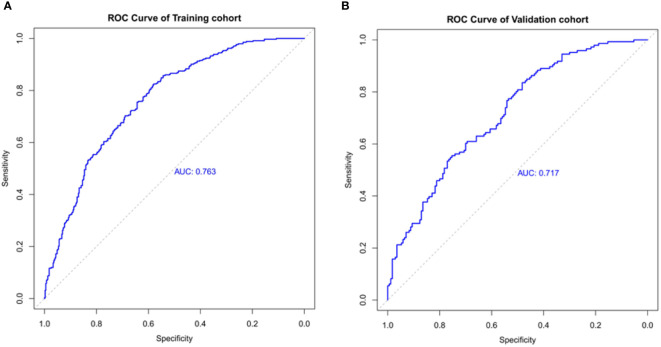
The nomogram model predicted the receiver operating characteristic curve of carotid atherosclerosis in patients with T2DM. **(A)** The area under the curve of the training cohort is 0.763. **(B)** The area under the curve of the validation cohort is 0.717.

**Figure 5:**
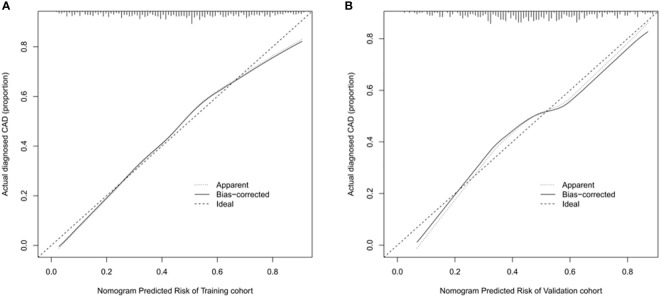
Calibration curve of nomogram model for predicting carotid atherosclerosis in patients with T2DM. **(A)** In the training cohort, B = 1000 repetitions, boot mean absolute error = 0.021, n = 733. **(B)** In the validation cohort, B = 1000 repetitions, boot mean absolute error = 0.032, n = 316. The X-axis represents the predicted risk of carotid atherosclerosis; Y-axis represents the actual diagnosed carotid atherosclerosis. Y-axis represents actually diagnosed carotid atherosclerosis. The diagonal dotted line represents the perfect prediction by the ideal model. The solid line represents the performance of the nomogram; the closer to the diagonal dotted line, the better the prediction.

**Figure 6:**
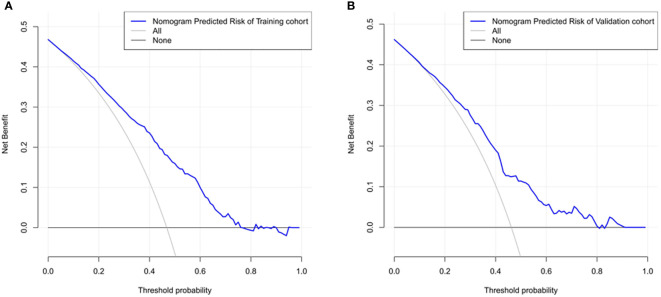
The nomogram model predicted the decision curve of carotid atherosclerosis in patients with T2DM. The y-axis measures the net return. The dotted line represents the CAD risk nomogram. The thin solid line represents the assumption that all patients are CAD. The thick solid line represents the assumption that no patients are CAD. **(A)** Decision curves for the training cohort show that using this nomogram predicts more benefit for CAD risk than intervening with the all-patient scenario or the no-intervention scenario if the threshold probabilities for patients and physicians are >2% and <76%, respectively. **(B)** Decision curves for the validation cohort show that using this nomogram to predict CAD risk adds more benefit than intervening with an all-patient regimen or a no-intervention regimen if the threshold probabilities for patients and physicians are >3% and <80%, respectively.

The ROC curve was used to assess the predictive accuracy of the nomograms. The results showed that the area under the ROC curve for the training cohort was 0.763 (95% CI = 0.73–0.80), and that of the validation cohort was 0.717 (95% CI = 0.66–0.77). The C- index of the training and validation cohorts were 0.76 and 0.72. The abovementioned results indicate that the nomogram has an excellent predictive effect on CAD (**Figure 4**). Next, a calibration curve was used to evaluate the deviation between the nomogram’s predicted results and actual values. The predicted results showed good agreement for both the training and validation cohorts (**Figure 5**). DCA curves were then used to assess the clinical usefulness of the nomograms. The results showed that in the training cohort, using this nomogram to predict CAD risk was more useful than the all-intervention or no-intervention methods if the threshold probabilities for patients and physicians were >2% and <76%, respectively. The decision curves show that in the validation cohort, CAD risk prediction with our nomogram is more informative than the all-intervention or no-intervention scheme when the threshold probabilities for patients and physicians are >3% and <80%, respectively (**Figure 6**). These results suggest that nomograms have excellent predictive power for CAD.

## Discussion

The nomogram model is a reliable statistical tool and is widely used with diagnostic prediction models of diabetic complications (13, 17). It uses a very intuitive graphical representation, which interprets risk models very simply and easily (18, 19). In this study, a CAD risk prediction model for patients with T2DM was developed using nomograms. The model was validated by the ROC curve, calibration curve, and C index, and the predicted and observed values were found to be in general agreement, indicating that the nomogram prediction model in this study is reliable. DCA showed that the nomogram has clinical applications. In the model’s process, five risk factors, age, fatty liver, smoking, high-density lipoprotein, and low-density lipoprotein, were screened and identified as risks of CAD in T2DM. According to the nomogram model, age was the largest risk factor among the five factors, followed by LDL-C and HDL-C, fatty liver, and smoking history. In addition, age, NAFLD, and OGTT3H were identified as independent risk factors for CAD in T2DM.

Most risk assessment methods for the development of CAD in T2DM include risk factor analysis. A study conducted in 2020 reported that age, gender, history of hypertension, coronary artery disease, and diabetes are risk factors for carotid atherosclerotic plaque formation (20). The risk factors for CAD in T2DM are similar to common risk factors for stroke in the Chinese population (21). Moreover, advanced age, male, lower education, hypertension, diabetes, passive smoking, and high LDL-C levels are independent risk factors for early atherosclerosis (22). From the results of these studies, several risk prediction models have been designed to assess the risk of carotid atherosclerosis disease in T2DM. However, limitations of these studies, such as individualized differences in populations, have led to a lack of simple and intuitive tools to facilitate the use of these models, so few of them have been applied in clinical practice. In our study, we developed the first nomogram describing CAD risk factors in T2DM. We performed an internal validation with the area under the ROC curves of 0.763 and 0.717 for the training and validation cohorts, respectively. The decision curves of the training cohort showed that using this nomogram to predict CAD risk in the present study was more favorable than all-patient intervention scenarios or no-intervention scenarios if the threshold probabilities for patients and physicians were 2% and 76%, respectively. Also, the DCA of the validation cohort was shown to be clinically useful.

The nomogram developed in this study allowed direct prediction and visual analysis of factors that greatly affect the risk of CAD in T2DM. Moreover, age is an unavoidable factor in many chronic diseases. According to a cohort study including 318,083 patients with T2DM from Sweden, age is greatly associated with T2DM with cardiovascular and mortality risk, which is consistent with the findings of our study (23). The possible mechanism for this is that the physiological and endocrine functions of the body diminish with age, leading to atherosclerosis; furthermore, older people tend to undergo physiological and structural changes in their blood vessels, thus reducing nitric oxide utilization and leading to increased production of angiotensin (24). Meanwhile, advanced age is an immutable risk factor for diabetes (25). Advanced age leads to the aging of pancreatic β-cells, resulting in defective insulin secretion and decreased glucose sensitivity (26); hyperglycemia is a risk factor for atherosclerosis (27). Persistent hyperglycemia causes changes in most cells in the vascular tissue, which accelerates atherosclerosis (28). Therefore, advanced age further affects blood glucose and aggravates atherosclerosis.

Our study also showed that NAFLD is strongly associated with the development of CAD in patients with T2DM. A study involving 8020 patients showed that persistent NAFLD was associated with an increased risk of developing subclinical CAD (29). Also, NAFLD is greatly associated with diabetes (30), further contributing to the development of CAD. The possible mechanism is that both NAFLD and T2DM are associated with a state of systemic hypo-inflammation, which may encourage atherosclerosis through the secretion of various cytokines such as interleukin-6, interleukin-1, tumor necrosis factor-α, and acute phase proteins (C-reactive protein, fibrinogen, and fetal protein-A) (31).

Smoking is still a major health hazard that significantly affects the morbidity and mortality of the cardiovascular disease. All stages of atherosclerosis are affected by it (32). The increased risk of cardiovascular disease in patients with diabetes is partly associated with a high prevalence of other cardiovascular disease risk factors. Therefore, the management of modifiable cardiovascular disease risk factors can minimize the risk of vascular complications in patients with diabetes (33).

The pathological process leading to atherosclerosis is usually associated with elevated LDL-C concentrations, which alter cell permeability and progressively affect the arterial wall (34). HDL-C may reduce the risk of cardiovascular events (35, 36). In addition, a large body of preclinical and mechanistic evidence suggests that HDL has an antidiabetic function and improves glycemic control by increasing insulin sensitivity and β-cell function (37). This is consistent with our study.

Therefore, it was essential to apply five risk predictors in our model. Despite the good performance of our nomogram, the study has some possible limitations. First, it was a retrospective study. The data collected did not provide information on other risk factors for diabetes, such as lifestyle factors, including chronic high-sugar diet or lack of exercise. Second, only internal validation was used. Third, all patients were from a single center with a limited sample size. Differences in ethnicity were not taken into account, which is not a good representation of the whole population. Finally, our collection had data with some missing values, which could introduce selection bias if participants with incomplete records from the full case study were completely excluded from model building. Therefore, we used multiple interpolations to replace missing values in the analysis. In the future, it is hoped that our study can be a collaborative effort of multiple centers to collect as many variables as possible to continuously test and modify predictive models in clinical practice.

In conclusion, we identified three CAD associated independent risk factors in T2DM, including age, NAFLD, and OGTT3H. Meanwhile, we screened and visualized five clinical indicators closely associated with the occurrence of CAD in T2DM using LASSO and logistics regression, which can be used as a clinical tool for clinicians to perform personalized screening by validating their model accuracy and good clinical usefulness.

## Data availability statement

The original contributions presented in the study are included in the article/**Supplementary Material**. Further inquiries can be directed to the corresponding author.

## Ethics statement

The studies involving human participants were approved by by the Ethics Committee of the Zhongnan Hospital of Wuhan University (ethical approval code: 2022167K). Written informed consent from the participants was not required to participate in this study in accordance with the national legislation and the institutional requirements.

## Author contributions

XF wrote the manuscript, performed data analysis, and prepared **Figures 1**-**5**; LR and YXi collected the data and prepared **Tables 1**-**2**; YXu designed the entire study, provided financial support, and supervised the whole process. All authors contributed to the article and approved the submitted version.
