# Supplementary figures and images for: Development and validation of a nomogram for evaluating the incident risk of carotid atherosclerosis in patients with type 2 diabetes
Source: Front Endocrinol (Lausanne). 2023 Feb 16;14:1131430. doi: 10.3389/fendo.2023.1131430 (PMC9978405; doi:10.3389/fendo.2023.1131430)

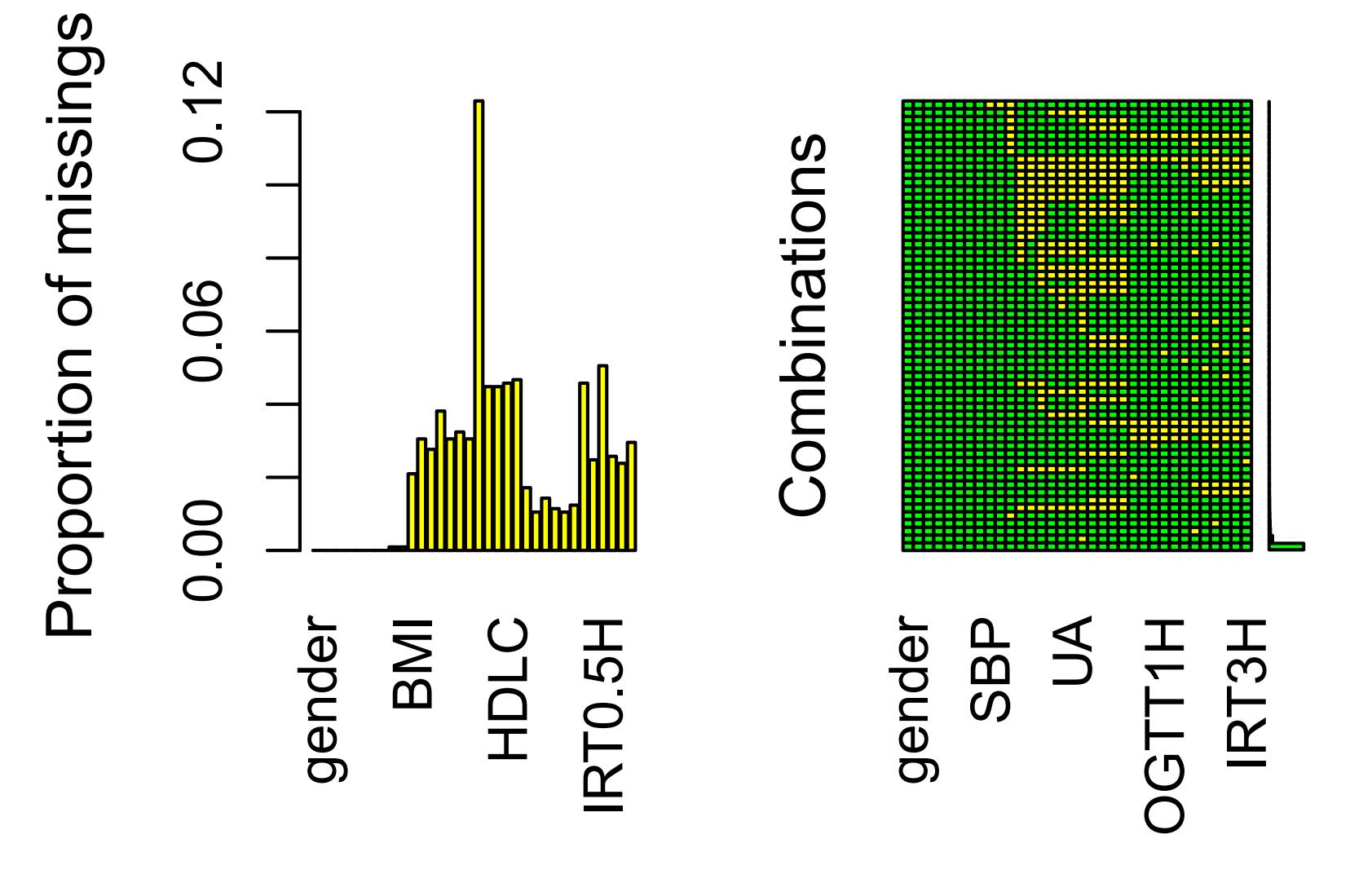

Supplement: Supplementary file 1 [file Image_1.jpeg]
